# Machine Learning Models for Predicting Mortality in Critically Ill Patients with Sepsis-Associated Acute Kidney Injury: A Systematic Review

**DOI:** 10.3390/diagnostics14151594

**Published:** 2024-07-24

**Authors:** Chieh-Chen Wu, Tahmina Nasrin Poly, Yung-Ching Weng, Ming-Chin Lin, Md. Mohaimenul Islam

**Affiliations:** 1 Department of Healthcare Information and Management, School of Health and Medical Engineering, Ming Chuan University, Taipei 111, Taiwan; ms1100663@ms1.mcu.edu.tw (C.-C.W.); kimweng@mail.mcu.edu.tw (Y.-C.W.); 2 Graduate Institute of Biomedical Informatics, College of Medical Science and Technology, Taipei Medical University, Taipei 110, Taiwan; d610108004@tmu.edu.tw; 3 Taipei Neuroscience Institute, Taipei Medical University, Taipei 110, Taiwan; 4 Department of Neurosurgery, Shuang Ho Hospital, Taipei Medical University, Taipei 110, Taiwan; 5 Department of Outcomes and Translational Sciences, College of Pharmacy, The Ohio State University, Columbus, OH 43210, USA

**Keywords:** acute kidney injury, sepsis, kidney disease, machine learning, algorithm bias

## Abstract

While machine learning (ML) models hold promise for enhancing the management of acute kidney injury (AKI) in sepsis patients, creating models that are equitable and unbiased is crucial for accurate patient stratification and timely interventions. This study aimed to systematically summarize existing evidence to determine the effectiveness of ML algorithms for predicting mortality in patients with sepsis-associated AKI. An exhaustive literature search was conducted across several electronic databases, including PubMed, Scopus, and Web of Science, employing specific search terms. This review included studies published from 1 January 2000 to 1 February 2024. Studies were included if they reported on the use of ML for predicting mortality in patients with sepsis-associated AKI. Studies not written in English or with insufficient data were excluded. Data extraction and quality assessment were performed independently by two reviewers. Five studies were included in the final analysis, reporting a male predominance (>50%) among patients with sepsis-associated AKI. Limited data on race and ethnicity were available across the studies, with White patients comprising the majority of the study cohorts. The predictive models demonstrated varying levels of performance, with area under the receiver operating characteristic curve (AUROC) values ranging from 0.60 to 0.87. Algorithms such as extreme gradient boosting (XGBoost), random forest (RF), and logistic regression (LR) showed the best performance in terms of accuracy. The findings of this study show that ML models hold immense ability to identify high-risk patients, predict the progression of AKI early, and improve survival rates. However, the lack of fairness in ML models for predicting mortality in critically ill patients with sepsis-associated AKI could perpetuate existing healthcare disparities. Therefore, it is crucial to develop trustworthy ML models to ensure their widespread adoption and reliance by both healthcare professionals and patients.

## 1. Introduction

Acute kidney injury (AKI) is a common public health threat worldwide, affecting one in five adults and one in three children [1]. The risk of AKI is even higher among patients with sepsis, playing a critical role in 40–50% of cases [2]. Previous studies have highlighted that sepsis increases the likelihood of in-hospital mortality by six- to eight-fold [3,4]. However, the timely identification and careful management of high-risk AKI patients may reduce mortality risk [5,6]. Recent technological advancements paved the way for developing automated real-time monitoring tools, facilitating appropriate clinical interventions [7,8,9]. 

In recent decades, machine learning (ML) has emerged as a pivotal tool for predicting disease onset and effectively managing medical conditions [10,11,12]. By leveraging extensive clinical datasets, comprising patient demographics, previous drug and disease history, organizational factors, lifestyle variables, and biomarkers [13], ML algorithms can correctly identify patterns and risk factors associated with conditions, such as AKI [14,15,16]. Existing algorithms can analyze complex interactions among various factors, facilitating the development of predictive models that assess an individual’s likelihood of developing AKI [17]. ML models also hold promise in personalized treatment plans by stratifying individual responses to various interventions, optimizing strategies for disease management, and ultimately improving patient outcomes [18,19,20]. 

However, developing fair ML models for stratifying high-risk AKI patients is crucial to minimize potential biases and disparities in healthcare outcomes. There is a dearth of prior research examining the fairness (e.g., testing ML performance in various racial and ethnic backgrounds) of ML models, a factor critical for ensuring equitable treatment and accurate classification across diverse demographic groups. This study aims to fill this gap by evaluating the performance of ML models in predicting mortality in patients with sepsis-associated AKI using insights gleaned from previously published research. Ensuring fairness in ML for mortality prediction, healthcare systems can offer more equitable and precise care to individuals, regardless of their racial and ethnic background or contextual orientations. Moreover, this study also provides strategies for reducing bias and developing fair models within real-world clinical settings. 

## 2. Methods

### 2.1. Search Strategy

A systematic search was conducted across popular electronic databases such as PubMed, Web of Science Core Collection, and Scopus between 1 January 2000 and 1 February 2024. The following keywords were used to retrieve all relevant articles: “machine learning” AND “sepsis-associated acute kidney injury” AND “mortality”. This search strategy underwent validation by an expert and involved the development of structure search strategies. Language restriction was not applied during the search process. The bibliographies of retrieved studies were further scrutinized to identify additional relevant studies for inclusion. 

### 2.2. Eligibility Criteria

Studies were only eligible for inclusion if they met the following criteria: (i) described ML algorithms, (ii) focused on mortality in patients with sepsis-associated AKI as the outcome of interest, (iii) reported the predictive performance of ML models, and (iv) were clinical studies. Studies were excluded if they (i) were not written in English or (ii) were reviews, editorials, letters, or conference abstracts. No specific study design or setting was prioritized in the inclusion criteria.

### 2.3. Study Selection

Two reviewers (M.M.I. and C.C.W.) independently screened the titles and abstracts to identify relevant studies for inclusion and exclusion. Eligible articles underwent full-text review, with duplicate studies removed. The same two reviewers conducted full-text screening if the studies met the inclusion criteria. Any discrepancies during the screening process were resolved through discussions between the two reviewers, reaching a final consensus.

### 2.4. Risk of Bias Assessment

The same two reviewers independently utilized the prediction model risk of bias assessment tool (PROBAST) to assess the methodological quality of each included ML model study [21]. This tool is specifically designed to assess the risk of bias and the applicability of diagnostic and prognostic prediction model studies. It is structured around four domains: participants, predictors, outcome, and analysis, which are crucial for evaluating the reliability and relevance of included studies. PROBAST rigorously assesses the methodologies to identify potential biases that might influence outcomes [22]. This tool is highly regarded for its systematic, structured method of assessing the integrity and clarity of the prediction model [23].

### 2.5. Data Extraction

A table was generated to record all relevant data. The extracted information was independently collected and cross-checked by the same two authors. Any discrepancies were resolved through consensus based on predefined criteria. All data items were collected according to the Cochrane guidance for data collection and the Critical Appraisal and Data Extraction for Systematic Reviews of Prediction Modelling Studies (CHARMS) checklist [24]. This tool is used to assist in the appraisal and extraction of relevant data from prediction model studies for systematic reviews [25]. This checklist guides reviewers through a comprehensive assessment of study methodologies, focusing on key aspects such as study participants, predictors, outcomes, and statistical analysis methods [26]. We extracted the following information from each study: first author, publication year, country, total number of patients, mean age, study design, AKI definition, patient inclusion and exclusion criteria, data partitioning method, name of ML model, internal and external validation details, and predictive performance metrics (including discrimination and calibration). If the included study reports multiple ML models, we separately recorded the predictive performance of each model.

### 2.6. Statistical Analysis

A descriptive summary was provided regarding the types of ML models used, the prediction of mortality in patients with sepsis-associated AKI, risk of bias assessment, and model validation. The results of ML studies were presented for each predicted outcome to illustrate the predictive performance of each type of ML model.

## 3. Results

### 3.1. Search Results

**Figure 1:**
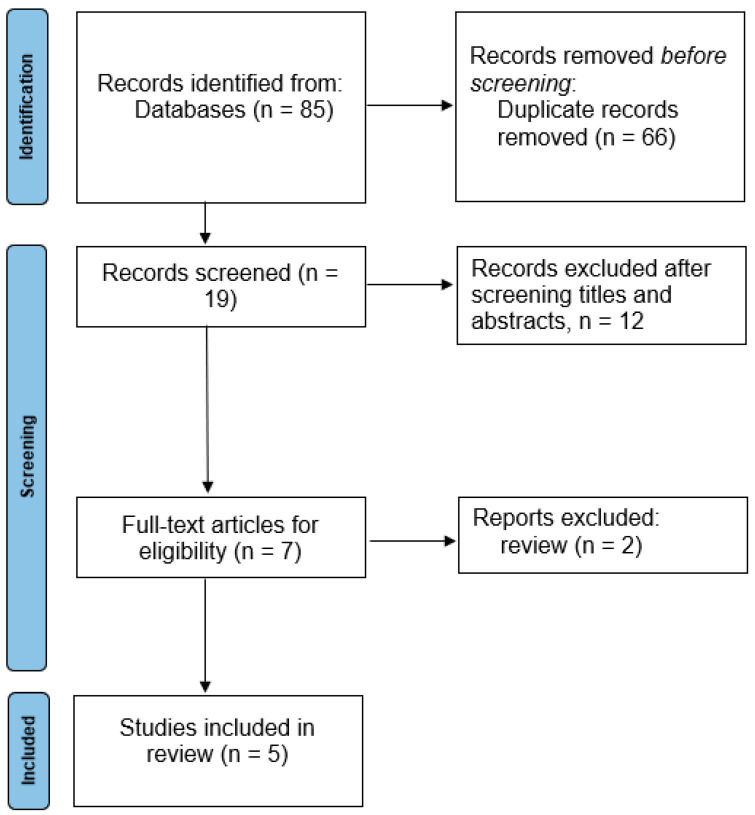
PRISMA flowchart diagram of study selection.

Initially, 85 studies were retrieved through the search process, followed by the exclusion of 66 duplicate studies. A total of 19 studies underwent initial screening, resulting in the exclusion of 12 studies based on title and abstract review. Of the remaining seven studies, a full-text evaluation was conducted, leading to the exclusion of two studies upon review. Finally, five studies met the criteria for inclusion in this systematic review [27,28,29,30,31] (Figure 1).

### 3.2. Study Characteristics

A total of 57,769 patients were enrolled across five studies. Table 1 presents the baseline characteristics of these studies. All studies utilized MIMIC data to develop and evaluate the performance of ML models. Luo et al. [27], however, employed data from the eICU Collaborative Research Database (eICU-CRD) to test their ML models. The majority (>50%) of patients across studies were male. While only two studies reported on race and ethnicity, a predominance of White patients was included [27,28]. Vital signs such as heart rate, respiratory rate, and blood pressure, along with laboratory tests including serum creatinine, platelets, white blood cell count (WBC), blood urea nitrogen (BUN), and urine outputs, were consistently assessed across all studies to predict mortality. Additionally, the Sequential Organ Failure Assessment (SOFA) score and Simplified Acute Physiology Score (SAPS II) were commonly utilized for outcome prediction in patients with severe sepsis-associated AKI. 

The studies included in the systematic review were predominantly conducted in ICU settings using Sepsis-3 criteria as the selection standard. Of the five studies reviewed, three utilized data partition methods for model development and evaluation, involving various approaches to dividing the dataset into training and testing subsets. Two studies employed cross-validation methods, iteratively training and testing the model on different data subsets to assess generalizability and performance. The proportion of missing data varied, and different imputation methods such as XGBoost, MiceForest, and K-nearest neighbor were employed. Notably, none of the studies tested their models across different racial groups, highlighting a significant gap in evaluating the models’ fairness and generalizability (Table 2).

### 3.3. Predictor Identification

Gao et al. [29] initially considered 51 original factors to predict mortality in patients with sepsis-associated AKI. They then applied ensemble stepwise feature ranking to identify the 11 most promising factors. Li et al. [30] collected 44 variables and identified 24 statistically significant variables associated with mortality using LASSO regression. Luo et al. [27] applied the XGBoost algorithm to assess the importance of various features in predicting mortality. For exploring the interpretability of these XGBoost models, the Shapley Additive Explanations (SHAP) method was also utilized. Yang et al. [31] initially conducted univariate regression analysis on all variables, excluding any with a *p*-value above 0.05. They then employed the Boruta algorithm, which relies on random forest techniques, to select the most important variables for predicting mortality. Finally, Zhou et al. [28] employed the recursive feature elimination (RFE) algorithm to pinpoint key variables for their ML models. 

### 3.4. Risk of Bias

**Figure 2:**
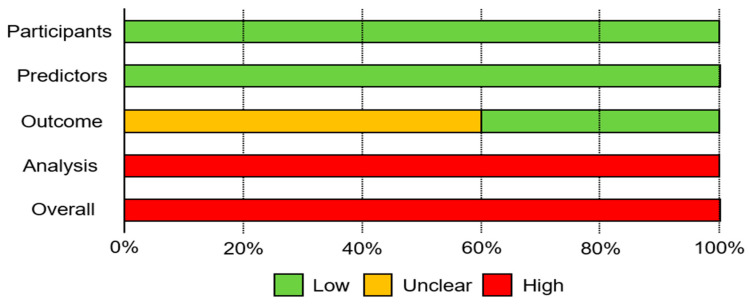
Overall risk of bias assessment of included studies using PROBAST.

Figure 2 presents the overall risk of bias assessment based on PROBAST criteria. All studies were rated as having a low risk of bias in both the participant and predictor domains. However, in the “outcome” domain, three studies (60%) received an unclear risk of bias rating due to insufficient information on the outcome definition and assessment. Additionally, all studies were rated as having a high risk of bias due to the absence of an external validation and evaluation of machine learning performance across diverse groups. Consequently, the overall risk of bias for the included studies was considered high, primarily due to the unclear ratings in the outcome domain and the lack of external validation.

### 3.5. Performance of Machine Learning Models

The predictive models demonstrated varying levels of performance, with area under the receiver operating characteristic curve (AUROC) values ranging from 0.60 to 0.87. The included studies developed and tested several popular machine learning (ML) models, including logistic regression (LR), random forest (RF), and extreme gradient boosting (XGBoost). All studies developed and validated the XGBoost model, which showed an area under the receiver operating characteristic curve (AUROC) ranging from 0.79 to 0.87 and an accuracy between 0.77 and 0.83. Random forest (RF) and logistic regression (LR) were also commonly used to predict mortality in patients with sepsis-associated AKI, achieving an average accuracy of 0.813 and an AUROC of 0.802. Table 3 presents the performance of these ML models in predicting mortality.

## 4. Discussion

This systematic review of ML models for predicting mortality in patients with sepsis-associated acute kidney injury (AKI) reveals several key findings and areas for future research. To our knowledge, this represents the first systematic review of ML studies aimed at predicting mortality in this patient population. The findings of this study show that included studies primarily conducted in ICU settings and utilized Sepsis-3 criteria for patient selection. Data partitioning and cross-validation methods were employed to develop and evaluate the models, with three studies using traditional partitioning techniques and two opting for cross-validation to ensure generalizability. 

Feature selection and imputation methods varied across studies, with XGBoost, MiceForest, and K-nearest neighbor, as these methods were crucial in handling missing data and identifying significant predictors of mortality. Vital signs, comorbidities, and laboratory tests emerged as critical prognostic indicators, aligning with clinical insights and reinforcing the importance of these variables in risk stratification. The included studies utilized widely used feature selection techniques such as SHAP, LASSO regression, and recursive feature elimination to identify key variables for their ML models. The performance of ML models varied across studies, with AUROC values ranging from 0.60 to 0.87. Notably, algorithms such as XGBoost, random forest (RF), and logistic regression (LR) consistently emerged as high-performing models. Despite the advancements, a significant gap was identified in the evaluation of these models across different racial groups. None of the studies included in the systematic review tested the performance of models in diverse racial populations, raising concerns about the fairness and generalizability of the findings.

Recent evidence underscores the importance of ensuring the fairness of ML models when predicting diseases such as AKI [32,33,34]. It is important to note that AKI affects individuals from diverse backgrounds, including various racial and ethnic backgrounds, socioeconomic status, sexual orientation, and geographical regions [35,36,37]. Without fair development, algorithms may disproportionately impact certain demographic groups, leading to inequitable healthcare outcomes. Algorithmic biases may also result in inaccurate predictions or misdiagnoses of high-risk AKI, potentially causing inappropriate treatments or delays in care. However, developing fair ML models ensures all individuals receive accurate assessments and appropriate interventions, regardless of their demographic characteristics [38,39].

Ethical concerns are increasingly raised regarding the differential impact that models may exert on under-represented communities [40,41,42]. Therefore, there is widespread acknowledgment of the urgent need for fairness in AI, especially within healthcare ML models [43]. With AKI prevalence higher among minority groups, it is essential to develop fair ML models using diverse and representative datasets. Continuous monitoring and testing of the ML model performance across various demographic groups are essential for identifying and effectively addressing relevant biases. Indeed, including individual, organizational, and community factors in the model development and validation processes can offer valuable insights into fairness considerations [33,44,45]. To ensure algorithms’ accountability and trustworthiness, transparent documentation of the model’s decision-making process and regular audits are recommended.

Strengths and Limitations:

Our study has several strengths. Firstly, it is the first systematic review assessing the fairness of ML models in predicting mortality among patients with sepsis-associated AKI. Secondly, our findings also elucidate commonly utilized ML models, offering valuable insights for researchers embarking on similar investigations. Nevertheless, our study has limitations. Our study has several limitations that could affect the robustness and applicability of our findings. Firstly, it is based on a small sample size, including only five existing studies, which may reduce the statistical power and comprehensive nature of our analysis. More studies could have broadened the scope and depth of our assessment. Secondly, the use of a single dataset across all studies limits the generalizability of our conclusions, as results might not be replicable with different data. Lastly, the performance of the ML models, while satisfactory in predicting mortality, is drawn from data with limited racial and ethnic diversity. This lack of representation could potentially perpetuate health disparities, suggesting a need for more diverse datasets to ensure broader applicability and equity in healthcare outcomes.

Future Direction:

To enhance the robustness and relevance of future studies, several steps should be taken based on the current limitations.

(a) External Validation:

One significant research gap is the absence of external validation. Most studies included in this review used the same dataset for both developing and evaluating the model. Future research should focus on validating machine learning models with external datasets to ensure their robustness and applicability across various clinical settings.

(b) Racial and Ethnic Diversity:

None of the included studies evaluated their models in diverse racial and ethnic groups, which raises concerns about fairness and generalizability. Future research should focus on developing and testing models that can be applied across various populations to help reduce health disparities.

(c) Advanced Imputation Techniques:

Although various imputation methods were used in the included studies, the development and application of more advanced techniques could significantly improve model accuracy, especially in datasets with high levels of missing data. Each study should report the percentage of missing data to assist researchers in future studies.

(d) Comprehensive Feature Selection:

Although the included studies utilized popular feature selection algorithms, future research should explore more comprehensive and innovative techniques to identify novel prognostic indicators. Integrating clinical expertise with advanced statistical methods can lead to more reliable predictors of mortality among sepsis patients with AKI.

(e) Longitudinal Data:

Using longitudinal data can enhance the predictive power of machine learning models for predicting mortality among sepsis patients with AKI. Future research should aim to develop models that incorporate and analyze temporal trends in patient data.

By addressing these gaps, future research can enhance the development of ML models that are not only accurate but also fair and generalizable, ultimately improving clinical outcomes for patients with sepsis-associated AKI. There is a need for research on the integration of ML models into clinical workflows; therefore, future studies should assess the feasibility, acceptance, and impact of these models in real-world clinical settings to facilitate their adoption.

## 5. Conclusions

In this study, we evaluated ML models to predict mortality in patients with sepsis-associated acute kidney injury (AKI), a condition noted for its high morbidity and mortality rates. Our findings show that ML models such as logistic regression, random forest, and extreme gradient boosting (XGBoost) delivered promising results. These outcomes highlight the efficacy of advanced data-driven methods in improving predictive accuracy, essential for timely interventions and management in septic patients. Key feature selection methods like Shapley Additive Explanations (SHAP), LASSO regression, and recursive feature elimination (RFE) were crucial in optimizing these models, underscoring the importance of selecting clinically relevant variables to maximize the ML model’s benefits in healthcare. Despite the robust performance of these ML models, included studies showed a significant risk of bias, potentially limiting the application of ML in medical diagnostics and planning. Particularly, included studies often excluded minority populations, which raises concerns about the models’ effectiveness across diverse socio-economic and racial groups. Future research should focus on enhancing the fairness of algorithms to ensure consistent and reliable predictions across all demographic groups.

## Figures and Tables

**Table 1:** Baseline chrematistics of included studies.

	Li et al. [30]	Luo et al. [27]	Yang et al. [31]	Zhou et al. [28]	Gao et al. [29]
	MIMIC IV (*N* = 8129)	MIMIC IV (*N* = 12,132)	MIMIC IV (*N* = 9158)	MIMIC IV (*N* = 16,154)	MIMIC IV (*N* = 12,196)
Demographic features					
Age	68.7 (57.2, 79.6)	68 (58–80)	67.0 (57.0–78.0)	67.7 (15.2)	67.0 ± 16.1
Sex, male, *n* (%)	4708 (57.9)	6920 (57.0)	5242 (57.2)	9318 (57.7)	6995 (57.4)
Body Mass Index (BMI)	N/A	N/A	29.2 (25.0–34.3)	30.9 (8.8)	29.6 ± 7.8
Race and Ethnicity					
White	N/A	8417 (69.3)	N/A	10,920 (67.6)	N/A
Black	N/A	1063 (8.7)	N/A	1733 (10.7)	N/A
Hispanic	N/A	375 (3.0)	N/A	538 (3.3)	N/A
Asian	N/A	297 (2.2)	N/A	377 (2.3)	N/A
Others	N/A	2010 (16.6)	N/A	2586 (16.0)	N/A
Comorbidities, *n* (%)					
Chronic pulmonary disease	2358 (29.0)	N/A	2352 (25.7)	N/A	3398 (27.9)
Peptic ulcer disease	261 (3.2)	N/A	268 (2.93)	N/A	N/A
Peripheral vascular disease	1143 (14.1)	N/A	1055 (11.5)	N/A	N/A
Myocardial infarction	1643 (20.2)	N/A	1522 (16.6)	N/A	2308 (18.9)
Cerebrovascular disease	1250 (15.4)	N/A	1200 (13.1)	N/A	2101 (17.2)
Diabetes	2566 (31.6)	N/A	2224 (24.3)	N/A	3669 (30.1)
Renal disease	1938 (23.8)	N/A	N/A	N/A	2394 (19.6)
Liver disease	1384 (17.0)	N/A	1548 (16.9)	3253 (20.1)	1611 (13.2)
Cancer	1108 (13.6)	N/A	1241 (13.6)	N/A	1611 (13.2)
Congestive heart failure	2831 (34.8)	N/A	2366 (25.8)	N/A	4010 (32.9)
Vital signs					
Heart rate (beats/minute)	86.1 (76.2–98.5)	87 (76–99)	105 (92.0–120)	106.1 (21.6) *	N/A
Mean arterial pressure (mmHg)	74.8 (69.6–81.3)	N/A	57.0 (50.0–63.0)	N/A	76.7 ± 10.1
Respiratory rate (beats/minute)	19.3 (16.9–22.4)	20 (17–24)	28.0 (24.0–32.0)	28.7 (6.7) *	19.9 ± 4.1
Body temperature (°C)	36.9 (36.6–37.3)	36.9 (36.6–37.3)	37.4 (37.0–38.0)	37.5 (0.8)	36.9 ± 0.7
SpO_2_ (%)	97.4 (95.9–98.7)	97 (95–99)	93.0 (90.0–95.0)	100 (100–100)	97.1 (2.2)
Systolic blood pressure (mmHg)	N/A	117 (106–130)	86.0 (78.0–94.0)	85.9 (16.4)	115.4 ± 15.1
Diastolic blood pressure (mmHg)	N/A	60 (53–68)	44.0 (38.0–50.0)		61.3 ± 10.4
Laboratory results					
Serum creatinine (mg/dL)	1.3 (0.9–2.1)	1.1 (0.7–1.9)	1.10 (0.80–1.60)	1.4 (0.9–2.5)	2.0 ± 1.7
Serum glucose (mg/dL)	155 (124–209)	N/A	101 (86.0–124)	143.0 (115.0–194.0)	179.5 ± 102.9
Serum chloride (mEq/L)	107 (103–111)	104 (100–108)	103 (99.0–106)	N/A	N/A
Serum calcium (mg/dL)	8.5 (8.0–9.0)	N/A	7.90 (7.40–8.40)	N/A	N/A
Hematocrit (%)	34.8 (30.7–39.7)	N/A		N/A	N/A
Hemoglobin (g/dL)	11.4 (10.0–13.1)	9.4 (8.3–10.6)	9.80 (8.30–11.3)	N/A	9.9 ± 2.2
Platelets (K/µL)	209 (151–282)	178 (112–270)	155 (105–218)	189.0 (135.0–257.0)	178.7 ± 104.7
Anion gap (mEq/L)	17.0 (14.0–20.0)	N/A	16.0 (13.0–19.0)	N/A	16.9 ± 5.1
WBC (K/µL)	14.6 (10.6–19.8)	11.3 (8.2–15.4)	14.8 (10.8–19.8)	13.5 (9.5–18.6)	16.2 ± 11.8
International normalized ratio (INR)	1.4 (1.2–1.7)	1.3 (1.2–1.6)	N/A	N/A	1.4 (1.2–1.7)
Partial pressure of oxygen (PaO_2_) (mmHg)	N/A	104 (84–133)	75.0 (46.0–103)	174.0 (104.0–321.0)	82.8 ± 54.3
Partial pressure of carbon dioxide (PaCO_2_) (mmHg)	N/A	40 (35–46)	47.0 (41.0–54.0)	100.0 (100.0–100.0)	49.3 ± 14.7
PaO_2_/FiO_2_ ratio	N/A	N/A	168 (100–248)	N/A	N/A
Ph	N/A	7.41 (7.36–7.45)	7.31 (7.24–7.36)	N/A	N/A
NLR ratio	N/A	N/A	7.88 (3.99–15.9)	N/A	N/A
PLR ratio	N/A	N/A	145 (77.8–281)	N/A	N/A
Bicarbonate (mmol/L)	24.0 (21.0–27.0)	24 (21–28)	24.0 (22.0–26.0)	N/A	20.9 ± 5.0
Serum sodium (mEq/L)	140 (137–143)	139 (136–143)	137 (134–140)	N/A	144.8 ± 5.8
BUN (mg/dL)	26.0 (17.0–42.0)	28 (17–46)	21.0 (16.0–32.0)	27.0 (18.0–45.0)	N/A
Serum potassium (mEq/L)	4.6 (4.2–5.1)	4.0 (3.7–4.4)	4.50 (4.10–5.00)	N/A	5.1 ± 0.9
Prothrombin time (s)	15.2 (13.2, 18.7)	N/A	N/A	N/A	18.4 ± 12.4
Partial thromboplastin time (PTT) (s)	34.2 (29.0–48.9)	32.5 (28.2–43.0)	N/A	34.4 (29.3–46.4)	33.9 (28.7–47.5)
Urine output (mL)	1295 (765–2000)	750 (410–1260)	1280 (815–1875)	1040.0 (537.0–1665.0)	1674.5 ± 1205.7
Treatments, *n* (%)					
RRT	592 (7.3)	8697 (11.2)	248 (2.71)	1633 (10.1)	N/A
Vasopressors use	785 (9.7)	22,573 (29.2)	N/A	5912 (36.6)	1873 (15.4)
Mechanical ventilation	7485 (92.1)	45,758 (59.1)	5326 (58.2)	9518 (58.9)	11,764 (96.5)
Severity scores of illnesses					
Sequential Organ Failure (SOFA) score	7 (5.0–10.0)	N/A	3.00 (2.00–5.00)	6.0 (4.0–9.0)	3.0 (2.0–5.0)
Simplified Acute Physiology Score (SAPS II)	42 (34–52)	N/A	39.0 (31.0–50.0)	42.0 (34.0–52.0)	42.1 ± 14.1
APSIII	N/A	N/A	52.0 (37.0–75.0)	N/A	N/A

Note: *N* = the number of participants in the study; * = standard deviation, N/A = Not Applicable.

**Table 2:** Summary of included studies on machine learning models for mortality prediction in sepsis-associated acute kidney injury.

Author	Publication Year	Setting	Selection Criteria	% of Missingness	Imputation Method	Feature Selection Method	Data Partition	External Validation	Model Testing in Different Racial Groups
Luo et al. [27]	2022	ICU	Sepsis-3	N/A	XGBoost	XGBoost	50:30:20	No	No
Li et al. [30]	2023	ICU	Sepsis-3	<20	MiceForest	LASSO	80:20	No	No
Yang et al. [31]	2023	ICU	Sepsis-3	<20	Random forest	Boruta algorithm	5-fold	No	No
Zhou et al. [28]	2023	ICU	Sepsis-3	N/A	K-nearest neighbor	Recursive feature elimination	80:20	Yes	No
Gao et al. [29]	2024	ICU	Sepsis-3	<25	MiceForest	Random forest	10-fold	No	No

N/A = Not Available.

**Table 3:** The performance of machine learning models for predicting mortality in patients with sepsis-associated acute kidney injury.

Study	Model	AUROC	Accuracy	Sensitivity	Specificity	Precision	F1-Score
Li et al. [30]	Decision Tree (DT)	0.585 (0.547–0.623)	0.737	0.378	0.812	0.425	N/A
	K-nearest Neighbor (K-NN)	0.601 (0.563–0.638)	0.793	0.367	0.783	0.429	N/A
	Support Vector Machine (SVM)	0.680 (0.643–0.717)	0.826	0.562	0.736	0.556	N/A
	Logistic Regression (LR)	0.730 (0.694–0.765)	0.822	0.608	0.754	0.572	N/A
	Random Forest (RF)	0.778 (0.745–0.812)	0.825	0.739	0.674	0.622	N/A
	Extremely gradient boosting (XGBoost)	0.794 (0.762–0.827)	0.832	0.793	0.752	0.660	N/A
Luo et al. [27]	Support Vector Machine (SVM)	0.754 (0.740–0.769)	N/A	N/A	N/A	N/A	N/A
	Random Forest (RF)	0.829 (0.819–0.840)	N/A	N/A	N/A	N/A	N/A
	Extremely gradient boosting (XGBoost)	0.848 (0.838–0.858)	0.789	0.743	0.791	N/A	N/A
Yang et al. [31]	Random Forest (RF)	0.849 (0.834–0.863)	N/A	N/A	N/A	N/A	N/A
	Logistic Regression (LR)	0.85 (0.836–0.864)	N/A	N/A	N/A	N/A	N/A
	Gradient Boosting (GBM)	0.865 (0.851–0.878)	N/A	N/A	N/A	N/A	N/A
	Extremely gradient boosting (XGBoost)	0.873 (0.860–0.886)	0.773	0.896	N/A	0.724	0.801
Zhou et al. [28]	Naive Bayes	0.76	0.68	0.74	0.67	0.37	0.49
	Support vector machine (SVM)	076	0.74	0.69	0.75	0.43	0.53
	Multi-layer perception (MLP)	0.79	0.73	0.70	0.73	0.41	0.52
	Logistic regression	0.79	0.73	0.71	0.74	0.41	0.52
	K-nearest Neighbor (KNN)	0.80	0.72	0.73	0.72	0.41	0.52
	Extremely gradient boosting (XGBoost)	0.81	0.77	0.68	0.79	0.46	0.55
	Gradient boosting decision tree (GBDT)	0.82	0.71	0.79	0.69	0.40	0.53
	Light gradient boosting (LightGBM)	0.82	0.74	0.75	0.74	0.43	0.55
	Adapting Boosting (AdaBoost)	0.82	0.79	0.65	0.83	0.51	0.57
	Random Forest	0.82	0.78	0.66	0.81	0.48	0.55
	Categorical Boosting (CatBoost)	0.83	0.75	0.75	0.75	0.44	0.56
Gao et al. [29]	Decision Tree	0.635 (0.611–0.659)	0.765	N/A	N/A	0.395	0.409
	K-nearest Neighbor	0.689 (0.663–0.714)	0.805	N/A	N/A	0.487	0.359
	SVM Linear	0.718 (0.691–0.743)	0.809	N/A	N/A	1.000	0.008
	Logistic Regression	0.756 (0.732–0.779)	0.816	N/A	N/A	0.568	0.258
	Naive Bayesian	0.764 (0.741–0.786)	0.795	N/A	N/A	0.446	0.354
	XGBoost	0.796 (0.774–0.817)	0.823	N/A	N/A	0.569	0.414
	Random Forest	0.798 (0.774–0.821)	0.832	N/A	N/A	0.661	0.372

N/A = Not Available.

## Data Availability

Not applicable.
